# Bilateral Medial Malleolus Stress Fractures in a High School Athlete Treated With Open Reduction Internal Fixation

**DOI:** 10.7759/cureus.18186

**Published:** 2021-09-22

**Authors:** Derek M Klavas, Brendan M Holderread, Jonathan Liu, Pedro E Cosculluela

**Affiliations:** 1 Department of Orthopedics and Sports Medicine, Houston Methodist Hospital, Houston, USA; 2 Department of Orthopedic Surgery, Brown University, Providence, USA

**Keywords:** medial malleolus, pediatric stress fracture, pediatric sports injury, bimalleolar ankle fracture, athlete

## Abstract

Pediatric medial malleolus stress fracture is a rare pathology and has limited data on management. The authors present a case of bilateral medial malleolus stress fracture treated with operative fixation followed by a course of immobilization and protected weight-bearing.

## Introduction

A medial malleolus stress fracture (MMSF) is a rare injury comprising only 4% of all stress fractures [1]. These injuries most commonly affect athletic individuals who engage in repetitive running/jumping activities (i.e. track and field athletes, military recruits, or basketball players) due to increased tibial stress [2-4]. Symptoms include pain with increasing duration of activity, swelling, and localized tenderness at the medial malleolus [1-4]. The pain may be poorly localized [5]. Plain radiographs may appear normal or in a vertical orientation originating from the tibial plafond [6]. The combination of pain correlating to the duration of the activity, tenderness over the medial malleolus, and vertical radiolucency originating from the tibial plafond should raise the treating physician’s suspicion for the diagnosis of MMSF [6].

Surgical intervention is often necessary to prevent long-term complications (non-union, fracture progression, and delays in healing) [3,4,7]. Surgically, open reduction internal fixation using cortical, cancellous, or cannulated screws demonstrate favorable results in patients with MMSF, but no standardized treatment algorithm exists [1,3].

Bilateral MMSFs are exceedingly rare, and management is challenging due to a lack of consensus treatment algorithms in these patients. The authors present a case of a 16-year-old male athlete with bilateral medial malleolus stress fractures managed successfully with surgical fixation followed by a period of immobilization and protected weight-bearing.

## Case presentation

**Figure 1:**
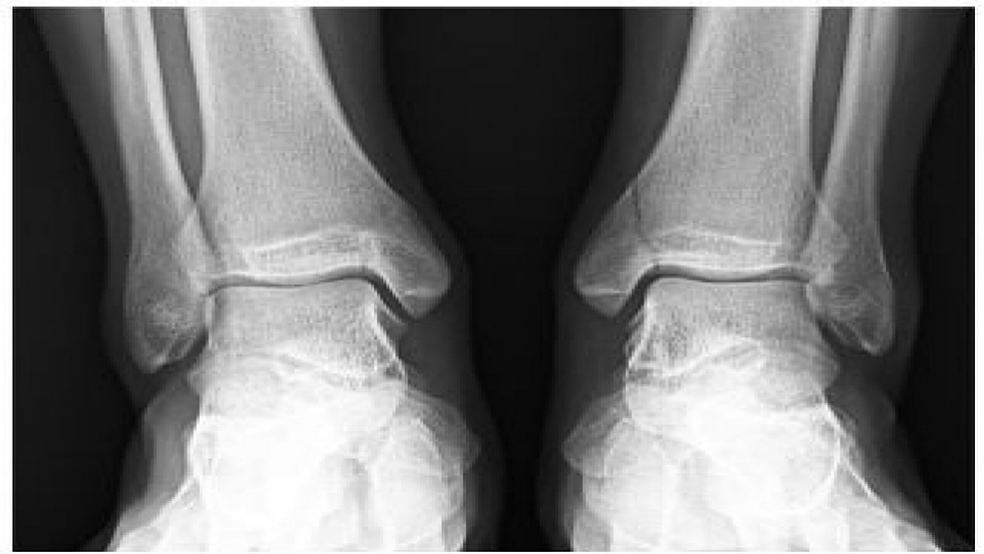
Pre-operative bilateral AP ankle radiograph demonstrating complete non-displaced medial malleolus stress fracture on the left, and incomplete medial malleolus stress fracture on the right. AP, anteroposterior.

A 16-year-old male high school athlete presented with the chief complaint of atraumatic left ankle pain and swelling one day after competing in a high-school varsity level football game. The patient reported mild, left medial-sided ankle pain for one month prior to presentation. The patient’s symptoms acutely worsened during competition, but he did not stop participating in the same game. Physical exam of a neurovascularly intact left foot and ankle revealed no postural abnormalities. There was swelling and tenderness localized to the medial malleolus, and pain with an external rotation stress test. Range of motion of the ankle, midfoot, and hindfoot was without restriction. Physical exam of a neurovascularly intact right foot and ankle revealed no postural abnormalities. There was no swelling, but palpable tenderness at the medial malleolus, and pain with external rotation stress. Bilateral standing anteroposterior (AP) ankle radiographs demonstrated a complete non-displaced medial malleolus stress fracture on the left and an incomplete medial malleolus stress fracture on the right (Figure 1).

The decision was made to proceed with surgery. This consisted of partially threaded 3.5 mm cannulated screws and one-third tubular applied in buttress fashion to the left distal medial tibia, and two partially threaded 3.5 mm cannulated screws inserted from medial to lateral parallel to the distal tibial plafond on the right. The patient was discharged home with a referral to a metabolic bone health specialist due to the chronic and atraumatic nature of their orthopedic injury. Differential diagnoses considered included osteoporosis, osteopenia, Paget’s disease, pathologic fracture, celiac disease, and chronic overuse/stress. Vitamin D 25 alpha-hydroxylase, parathyroid hormone, calcium, and alkaline phosphatase levels were all within normal limits. The dual-energy X-ray absorptiometry (DEXA) scan demonstrated no evidence of osteoporosis or osteopenia (Table 1). The clinical presentation and normal metabolic workup were suggestive of overuse as etiology.

**Table 1:** Metabolic bone workup. AP, anteroposterior; Alk Phos, alkaline phosphatase; PTH, parathyroid hormone; Vit D 25-OH, vitamin D 25 alpha-hydroxylase.

Laboratory workup	Result	Reference range
Vit D 25-OH (ng/mL)	47.2	20-150
Calcium (mg/dL)	9.9	8.6-10.6
Alk Phos (mol/L)	160	65-260
PTH (pg/mL)	16.0	13-65
Bone densitometry	Result (g/cm2)	T-score
AP spine (L1-L4)	1.15	−0.2
Bilateral femurs (mean)	1.19	0.8

**Figure 2:**
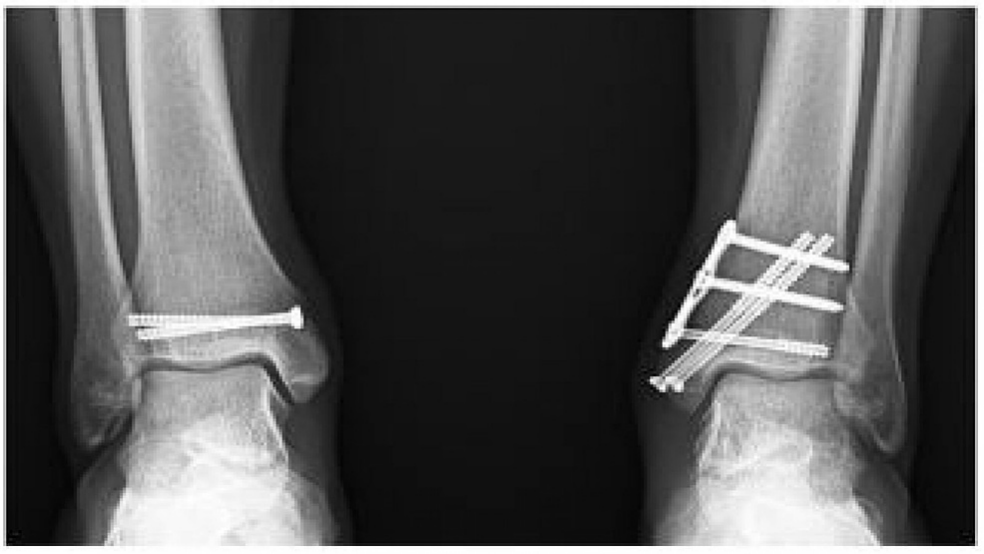
Three-month post-operative radiograph demonstrating routine healing of bilateral medial malleolus stress fractures.

Post-operatively, range of motion exercises of the ankle were started with physical therapy. The left lower extremity was kept non-weight bearing in a controlled ankle motion-boot for six weeks. After six weeks, the patient was transitioned to weight-bearing as tolerated on the left lower extremity. The right lower extremity was non-weight-bearing in the two weeks immediately following surgery, and was transitioned to weight-bearing as tolerated at two weeks. Bilateral ankle range of motion exercises were recommended in the two-week post-operative period and physical therapy with ankle fracture protocol was prescribed at two weeks post-operative follow-up. Three months post-operatively, follow-up radiographs demonstrated a completely healed right medial malleolus and near-complete healing on the left (Figure 2). The patient was cleared for a gradual return to sport and athletic shoe wear at this time.

At six months post-operative follow-up, radiographs demonstrated completely healed bilateral medial malleolus stress fractures with intact hardware. The patient reported no pain bilaterally at the medial ankle and hindfoot. The patient's physical therapy course was completed, and he had returned to full sprinting activities. Ten months post-operatively, the patient was participating in sports without restriction and the American Orthopedic Foot and Ankle Society (AOFAS) hindfoot score calculated by chart review was 100/100 at 10 months.

## Discussion

Medial malleolus stress fractures are seen most frequently in athletes and military recruits due to abnormal weight transmission and torsional forces [6]. Patients often present with a vague progression of pain and discomfort in the medial side of the distal tibia after training [7]. A triad for MMSF has been described with the presentation of pain during activities before an acute episode, a radiolucent vertical line from the tibial plafond, and point tenderness over the medial malleolus [6]. As seen with our patient, the initial symptoms may be mild and develop insidiously with continued activity and competition. MMSF is investigated with AP radiographs to assess for fracture lines and spurring on the tibia or talus [1]. Negative radiographs with high clinical suspicion should be further evaluated with MRI or CT imaging to establish the diagnosis [5,8]. A young athlete presenting with bilateral MMSF has been previously reported [9]. Percutaneous fixation using cannulated, double-threaded screws bilaterally has been reported previously in a young athlete presenting with bilateral MMSF previously [9]. However, the right-sided MMSF was not identified on the initial presentation and two separate operations were performed.

Most stress fractures can be managed non-operatively with a boot or cast immobilization and protected weight-bearing for six to eight weeks [3]. However, the literature suggests that early surgical treatment is associated with better outcomes and faster return to sports and non-surgical treatment may result in prolonged healing and recurrent stress fractures [10]. Kaeding et al. classify medial malleolar fracture as high risk (based on fracture location, medial malleolus and complete fractures were considered high risk) for non-union and recurrence and recommend that non-operative management should only be considered in cases where fracture lines are only detectable with MRI and non-evident on CT or X-ray [11]. The patients included in their study led them to the conclusion that low-risk patients should be strictly non-weight bearing and monitored closely for healing with a low threshold for surgery [11]. The patient in this case report would be classified as high-risk, due to location (medial malleolus), complete fracture on the left, and fracture visibility bilaterally on plain radiographs [11]. Anderson et al. recommend open reduction and internal fixation with a one-third tubular plate and 3.5-mm screws for complete medial malleolus fractures [3]. For incomplete or non-displaced fractures, percutaneous fixation should be considered for incomplete or non-displaced fractures due to less soft tissue trauma and shorter recovery times compared to open techniques [12]. In our unique case of bilateral MMSF of varying severity, the operative surgeon proceeded with plate fixation of the complete fracture of the left ankle and percutaneous fixation of the incomplete fracture of the right ankle.

Nguyen et al. recommend immobilization in a boot for six weeks for complete medial malleolus fractures after open reduction and internal fixation (ORIF) with early post-operative range of motion (ROM) exercises starting after two weeks [1]. Patients were gradually introduced to full activity and had excellent outcomes in regard to patient satisfaction, fracture union, and return to high-level sport [1]. Incomplete fractures should be immobilized and non-weight bearing for one to two weeks post-operatively with gradual weight-bearing and full activity starting at four to six weeks [13]. Patients should be reassessed at three months to confirm fracture union, at which point, return to sports can be considered with gradual training as tolerated [13]. The literature suggests that radiographic union of complete fractures post-ORIF occurs around four months compared to seven months for non-operative patients [10]. In their cohort of 16 professional soccer players, Nguyen et al. reported an average of four months post-operatively for a return to professional sport at the same level of activity [1]. Regarding our patient, the incidence of bilateral fractures did not prolong the recovery process and radiographs showed bilateral fracture union at three months post-operatively. At this point, our patient was cleared for a gradual return to sport and reports no complications at six months post-operatively.

Due to the unprecedented presentation of bilateral MMSF in a young athlete, laboratory evaluation for metabolic etiologies was necessary. Some studies have reported an association between low levels of 25-hydroxy vitamin D and stress fractures [14]. While others found that higher parathyroid hormone (PTH) levels may be more significant and indicate a subclinical vitamin D deficiency or resistance [14]. Additionally, relative energy deficiency in sport (RED-S) due to low energy availability (EA) may contribute to compromised physiological processes in athletes [15]. The inadequate energy intake relative to exercise expenditure is associated with stress fractures, menstrual irregularities in females, decreased libido, and other metabolic conditions [15]. The current literature recommends using surrogate markers including resting metabolic rate and validated questionnaires to screen for low EA in athletes who present with abnormal conditions without known etiology [15]. While the metabolic workup for our patient was unremarkable, it is important to consider the role of RED-S in abnormal fracture presentations and provide sports nutrition education and recommendations for the prevention of future complications.

## Conclusions

A medial malleolus stress fracture is a rare but well-documented injury in running and jumping athletes. A bilateral medial malleolus stress fracture is a rare clinical entity that has only been described in the literature on a case-report basis. The possibility of metabolic bone abnormality warrants referral to a bone health specialist. The patient was successfully treated with surgical fixation, demonstrated routine radiographic healing of the fractures post-operatively at six months, and reported no limitations impacting the quality of life by 10 months.
